# Comparison of 3D MRI with high sampling efficiency and 2D multiplanar MRI for contouring in cervix cancer brachytherapy

**DOI:** 10.2478/v10019-012-0023-1

**Published:** 2012-04-11

**Authors:** Primoz Petric, Robert Hudej, Peter Rogelj, Mateja Blas, Barbara Segedin, Helena Barbara Zobec Logar, Johannes Carl Athanasios Dimopoulos

**Affiliations:** 1 Department of Radiotherapy, Institute of Oncology Ljubljana, Ljubljana, Slovenia; 2 University of Primorska, Faculty of Mathematics, Natural Sciences and Information Technologies, Koper, Slovenia; 3 Institute of Oncology Ljubljana, Research Sector, Unit for Biostatistics, Ljubljana, Slovenia; 4 Metropolitan Hospital, Department of Radiotherapy, Athens, Greece

**Keywords:** cervix cancer, brachytherapy, contouring, MRI

## Abstract

**Background:**

MRI sequences with short scanning times may improve accessibility of image guided adaptive brachytherapy (IGABT) of cervix cancer. We assessed the value of 3D MRI for contouring by comparing it to 2D multi-planar MRI.

**Patients and methods:**

In 14 patients, 2D and 3D pelvic MRI were obtained at IGABT. High risk clinical target volume (HR CTV) was delineated by 2 experienced radiation oncologists, using the conventional (2D MRI-based) and test (3D MRI-based) approach. The value of 3D MRI for contouring was evaluated by using the inter-approach and inter-observer analysis of volumetric and topographic contouring uncertainties. To assess the magnitude of deviation from the conventional approach when using the test approach, the inter-approach analysis of contouring uncertainties was carried out for both observers. In addition, to assess reliability of 3D MRI for contouring, the impact of contouring approach on the magnitude of inter-observer delineation uncertainties was analysed.

**Results:**

No approach- or observer - specific differences in HR CTV sizes, volume overlap, or distances between contours were identified. When averaged over all delineated slices, the distances between contours in the inter-approach analysis were 2.6 (Standard deviation (SD) 0.4) mm and 2.8 (0.7) mm for observers 1 and 2, respectively. The magnitude of topographic and volumetric inter-observer contouring uncertainties, as obtained on the conventional approach, was maintained on the test approach. This variation was comparable to the inter-approach uncertainties with distances between contours of 3.1 (SD 0.8) and 3.0 (SD 0.7) mm on conventional and test approach, respectively. Variation was most pronounced at caudal HR CTV levels in both approaches and observers.

**Conclusions:**

3D MRI could potentially replace multiplanar 2D MRI in cervix cancer IGABT, shortening the overall MRI scanning time and facilitating the contouring process, thus making this treatment method more widely employed.

## Introduction

Image guided adaptive brachytherapy (IGABT) enables individualized irradiation, applying high doses to the target volume while respecting organs at risk (OAR) dose constraints.^1^,^2^ Accurate contouring of these regions is a precondition for treatment success and consistent reporting in brachytherapy as well as in external beam irradiation.^3^ Various imaging modalities have been employed in gynaecological IGABT.^4^–^23^ T2 weighted fast spin echo (T2w FSE) MRI currently represents the modality of choice due to its high soft tissue depiction quality.^16^–^28^ Favourable reports on diagnostic and dosimetric outcome of MRI based approach are reflected in encouraging clinical results.^18^–^24^,^29^,^30^

**FIGURE 1:**
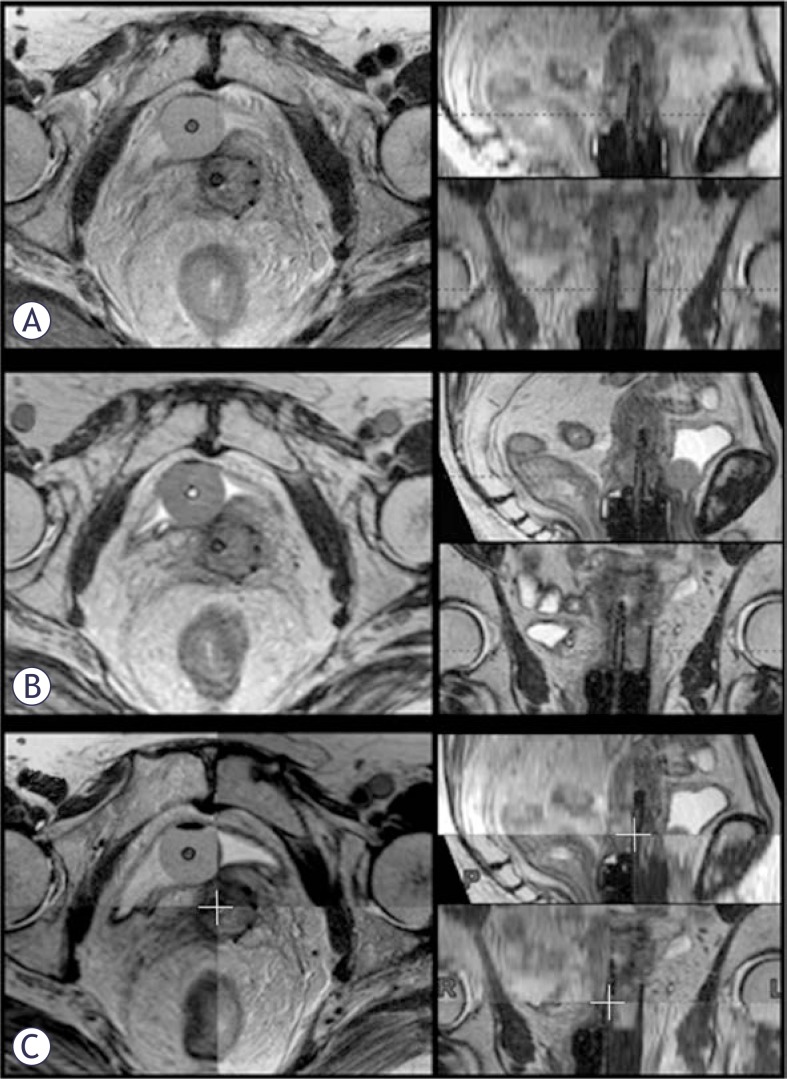
Post-insertion pelvic MRI. (A) Para-transverse 2D T2w FSE MR images were imported into the TPS (left). Para-sagittal and -coronal images were resampled from this data-set (right): due to the 3.9 mm slice thickness, their resolution is poor. (B) 3D MRI data-set was imported into the TPS. High resolution para-transverse (left), -sagittal and -coronal images (right) were resampled due to an isotropic voxel size of 1 mm. (C) Co-registration of the 2D para-transverse and 3D MRI data-sets.

However, limited access to MRI precludes its widespread adoption in this field. Until the role of low cost modalities (CT and US) is systematically evaluated, implementation of high resolution MRI sequences with short scanning times may make IGABT available to a wider population of patients. Currently, assessment of multiplanar post-insertion 2D T2w FSE MRI is required for accurate contouring.^1^,^2^,^27^ However, majority of treatment planning systems (TPS) do not enable import of non-resampled images in multiple planes. Classically, only (para)transverse images are imported and used for delineation, while other planes are resampled by the TPS from the (para)transverse set. Due to the slice thickness of 3–5 mm, the resampled images have poor resolution and are not useful for evaluation of patho-anatomical structures (Figure 1A). Non-resampled high-resolution (para)sagittal and (para)coronal 2D MR images have to be obtained in addition. As a result, 2D multiplanar MRI is characterized by long scanning time and the need for a separate DICOM-viewer to integrate findings from different planes during contouring.^1^,^2^,^27^,^28^ This approach is currently used at the Institute of Oncology Ljubljana. In addition, a 3D T2w FSE sequence with high sampling efficiency, 1 mm isotropic voxel size and large field of view (SPACE) is obtained at our department (Figure 1B). 3D MRI is co-registered with 2D para-transverse images in the TPS to reduce applicator reconstruction uncertainties.^31^ Due to the small voxel size, high resolution images in multiple planes can be resampled by the TPS from the 3D data-set (Figure 1C).

The aim of our study was to assess whether the 3D SPACE sequence could potentially replace multiplanar 2D MRI for contouring of high risk clinical target volume (HR CTV). By omitting multiplanar 2D MRI, the scanning time would be shortened. In addition, utilizing high-resolution images, resampled in multiple planes from the 3D data-set by the TPS, the separate DICOM viewer would no longer be required and fusion of multiple image series with its inherent uncertainties could be avoided.

## Patients and methods

The value of 3D MRI for contouring was evaluated by using the inter-approach and inter-observer analysis of volumetric and topographic contouring uncertainties. In addition, to assess reliability of 3D MRI for contouring, the impact of contouring approach on the magnitude of inter-observer delineation uncertainties was analysed.

To assess the magnitude of deviation from the conventional approach when using the test approach, the inter-approach analysis of contouring uncertainties was performed for both observers.

The study was carried out according to the Helsinki Declaration.

### Patients and tumours

Fourteen consecutive patients with biopsy proven cervix cancer (8 FIGO stage IIB and 6 stage IIIB), treated at our department with radical MRI-based IGABT between December 2006 and September 2007, were included. Mean tumour width, thickness and height, measured from the MRI at diagnosis, were 51 (Standard deviation (SD 11), 41 (SD 10) and 45 (SD 16) mm, respectively. Treatment consisted of 3D conformal CT-based external beam radiotherapy +/− concurrent chemotherapy, followed by 2 fractions of MRI-based pulsed dose rate IGABT. Details of our treatment strategy were presented elsewhere.^32^ First IGABT fraction from each case was used for analysis.

### Post-insertion MRI and image registration

Planning MRI was obtained after applicator insertion at a 1.5 T scanner (Siemens Magnetom Avanto© 2006, Siemens AG, Erlangen, Germany), using a pelvic surface phased-array coil. 2D T2w FSE images (slice thickness 3 mm, interslice gap 0.9 mm, in-plane pixel size 0.6 × 0.6 mm, field of view 20 × 20 cm, matrix size 288 × 320, echo time 98 ms, repetition time 5700 ms, flip angle 150°, acquisition time ≈12 minutes), were obtained in paratransverse (perpendicular to cervical canal), paracoronal and para-sagittal (parallel to cervical canal) plane. In addition, 3D isotropic T2w FSE sequence with high sampling efficiency (SPACE, a vendor-specific sequence) was performed (176 slices, iso-tropic voxel size of 1 mm, field of view 40 × 40 cm, matrix size 384 × 386, echo time 131 ms, repetition time 1500 ms, flip angle 150°, acquisition time ≈7 minutes). Para-transverse 2D images and 3D data set were transferred to the TPS (Brachyvision, version 8.5, Copyright© 1996–2008 Varian Medical Systems Inc., Palo Alto, USA) and co-registered, using shared DICOM coordinates. Manual registration corrections were applied where patient movement occurred between sequences. From the 3D data-set, para-transverse, para-coronal and para-sagittal images were resampled within the TPS to match the slice thickness and acquisition planes of the 2D images (Figure 1C).

### Contouring

HR CTV was outlined independently by 2 experienced radiation oncologists, respecting the GEC-ESTRO recommendations, using two different approaches.^1^ (1) *Conventional approach:* contouring on non-resampled para-transverse 2D MRI; during delineation, non-resampled para-sagittal and para-coronal 2D images were available on a separate DICOM viewer to assess topographical relations between the target, applicator and normal anatomical structures in 3 dimensions. This information was taken into account and integrated on para-transverse images in the TPS, where contouring was performed. (2) *Test approach:* contouring on para-transverse images, resampled from the 3D MRI; resampled high resolution para-sagittal and para–coronal images were available in the TPS contouring windows (Figure 1B), enabling direct incorporation of spatial information on patho-anatomical structures and interactive assessment of contours in all planes, without the use of an additional DICOM viewer. Contours, obtained in this study, were used for the purpose of analysis only, not for actual treatment. The interval between conventional and test contouring was at least one month.

### Volumetric and topographic analysis

Using a dedicated software tool (Contour Analysis Tool – CAT, version 2), developed at our departments, inter–approach (intra-observer) and inter-observer contouring variation was assessed for each observer and approach, respectively. In volumetric analysis, HR CTV sizes were compared. In addition, volumetric conformity index (VCI) was computed for pairs of HR CTVs as the ratio between the common and encompassing volume^33^, and compared within the inter-approach and inter-observer analysis. The common volume is defined as the intersection of two contoured volumes, while the encompassing volume is their union. In topographic analysis of inter-approach variations, the shortest 2D distance between conventional and test contour was calculated for each contour point on each slice for both observers. In inter-observer assessment, corresponding distances between delineations of the two observers were measured for both approaches. In order to identify eventual dependence of the variation magnitude on craniocaudal level of the contoured volume, the analysis was carried out for all slices and, in addition, for the following HR CTV levels: (1) caudal-most two, (2) mid-level, and (3) cranial-most two slices.

### Statistical analysis

Continuous numerical variables were presented as mean values and standard deviations. Inter-approach and inter-observer variation was assessed by calculating Bland-Altman limits of agreement^34^,^35^, and Lin’s concordance correlation coefficient.^36^,^37^ We used the bootstrap method to obtain a more reliable 95% confidence interval for the correlation coefficient. All analyses and graphs were performed using a statistical program R (version 2.11.1., R Foundation for Statistical Computing, Vienna, Austria).

## Results

No approach- or observer - specific differences in HR CTV sizes, VCI, or distances between contours were identified. The magnitude of topographic and volumetric inter-observer contouring uncertainties, as obtained on the conventional approach, was maintained on the test approach. This variation was comparable to the inter-approach uncertainties from both observers. Variation was most pronounced at caudal HR CTV levels in both approaches and observers. Detailed results of our analysis are presented below.

Using the conventional approach, mean HR CTVs of 32 (Standard deviation (SD) 10.5) cm^3^ and 31.8 (SD 10.8) cm^3^ were obtained for observer 1 and 2, respectively. Using the test approach, the respective HR CTV sizes were 33.2 (SD 11.0) cm^3^ and 30.4 (SD 11.2) cm^3^.

**FIGURE 2:**
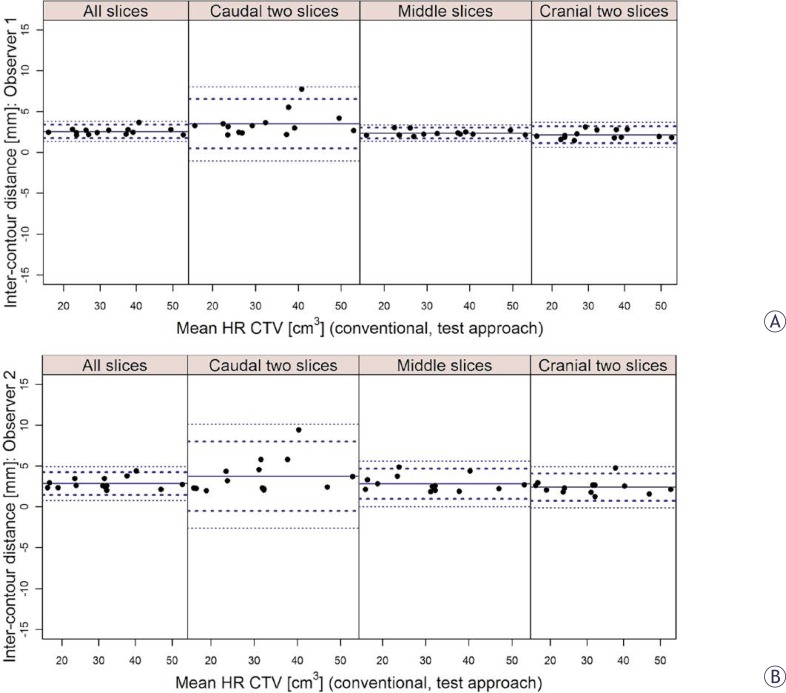
Bland-Altman inter-approach analysis of the distances between contours for observer 1 (A) and 2 (B) and for different levels of the contoured volume (all, caudal two, middle and cranial two slices). Full circles: mean values (mm) of the individual distances. Thick dotted lines: limits of agreement (mean ± 2 standard deviations). Thin dotted lines: 95% confidence limits.

Using the inter-approach Bland-Altman analysis, high agreement in HR CTV sizes was found for both observers (Table 1). A favourable mean VCI of 0.8 (SD 0.03) and 0.79 (SD 0.04) was obtained for observer 1 and 2, respectively. The results of the Bland-Altman analysis of the inter-approach distances between contours were comparable for both observers and are presented in Table 2 and Figure 2. When averaged over all slices of the HR CTV, mean inter-approach distances were 2.6 mm (SD 0.4 mm) for observer 1 and 2.8 mm (SD 0.7 mm) for observer 2. Inter-approach contouring variation was most pronounced at the caudal level of the HR CTV (observer 1: 3.5 mm, SD 1.5 mm; observer 2: 3.7 mm; SD 2.1 mm). At the mid and cranial levels of the HR CTV, the inter-approach distances between contours were smaller (Table 2, Figure 2).

**FIGURE 3:**
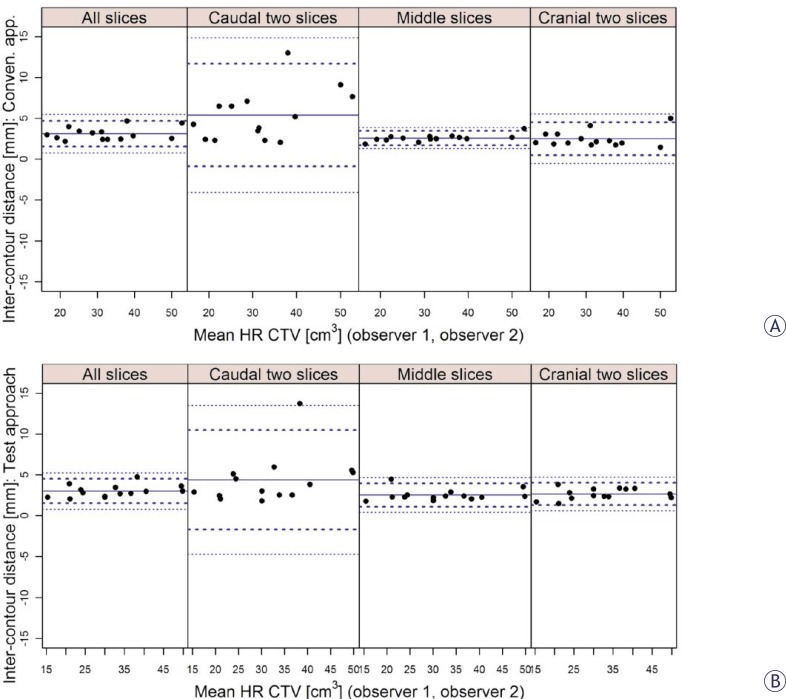
Bland-Altman inter-observer analysis of the distances between contours for conventional (A) and test (B) approach and for different levels of the contoured volume (all, caudal two, middle and cranial two slices). Full circles: mean values (mm) of the individual distances. Thick dotted lines: limits of agreement (mean ± 2 standard deviations). Thin dotted lines: 95% confidence limits.

On the inter-observer analysis, high agreement in HR CTV sizes was found for both approaches (Table 1). Favourable mean VCI on conventional approach of 0.76 (SD 0.05) was maintained on test approach (mean VCI: 0.75; SD 0.05). The results of the Bland-Altman analysis of the inter-observer distances between contours were comparable for both approaches and are presented in Table 2 and Figure 3. When averaged over all slices of the HR CTV, mean inter-observer distances between contours of 3.1 mm (SD 0.8 mm) were found on conventional and 3.0 mm (SD 0.7 mm) on test approach. Similar to the inter-approach analysis, highest inter-observer distances were obtained at the caudal level of the HR CTV on the inter-observer assessment and were comparable for both contouring approaches (conventional: 5.4 mm, SD 3.1 mm; test: 4.4 mm, SD 3.0 mm). At the mid and cranial levels of the HR CTV, the inter-observer distances were smaller (Table 2, Figure 3).

## Discussion

The dose conformity in IGABT exceeds any other radiotherapy modality and even minimal contouring variation can result in significant uncertainties of optimized dose distribution.^37^ This may have important clinical consequences and compromise treatment recording and reporting, undermining overall IGABT efficacy. One of the potential sources of systematic contouring variations is the choice of imaging modality.

High resolution 3D T2w FSE isotropic MRI with high sampling efficiency, small voxel size, large field of view and sufficient signal to noise ratio was introduced for diagnostic imaging of the pelvis and other anatomical regions, potentially improving the diagnostic possibilities of MRI.^38^–^46^ Nevertheless, it seems that further improvements of 3D MRI are required before it could be considered as a replacement for 2D multiplanar imaging in local staging.^38^,^40^ As far as imaging for gynaecological IGABT treatment planning is concerned, 2D T2w FSE multi-planar MRI is currently considered the modality of choice.^1^,^2^ Results of the MRI-based IGABT have been published by several groups, demonstrating improved chance of cure and reduced morbidity rates when compared to conventional radiography-based method.^20^–^23^,^47^ Nevertheless, in spite of these favourable reports, widespread utilization of MRI and IGABT remains impeded by limited resources.^48^–^50^ The innovative approach to 3D MRI-based contouring, evaluated in our study, may make IGABT more easily employed. By omitting post-insertion multi-planar 2D MRI and by using 3D SPACE sequence both for applicator reconstruction and contouring, the MRI scanning time was reduced from approximately 19 to 7 minutes in our study. The achieved reduction is comparable to other strategies that could be proposed to improve IGABT availability (i.e. combination of MRI for the first application and CT, ultrasound or radiography for subsequent application(s)). Importantly, by applying a single sequence, uncertainties due to eventual patient motion between sequences were reduced. However, the introduction of a new imaging approach could result in sequence-specific observer’s interpretation of findings and systematic deviations from our standard technique. No such deviations could be found in our study. In addition, no observer-specific deviations were identified and the magnitude of intra-observer uncertainties was comparable to inter-observer variation.

VCI is one of the commonly used measures of volumetric similarity between delineated objects. Poor agreement is indicated by a low VCI. As the agreement increases, VCI approaches 1. Small differences in HR CTV sizes (Table 1) and favourable results on VCI, obtained in our study, indicate high level of volumetric agreement between the conventional and test delineations. An inter-approach VCI of around 0.8 compares favourably with results of similar studies both in the field of EBRT and BT.^51^–^57^ In addition, high inter-observer VCI of the conventional 2D MRI approach was maintained for 3D MRI-based contouring. However, the sensitivity of VCI to contouring deviations increases with decreasing size of analysed volumes: contouring deviation of a given absolute magnitude will be reflected in a more favourable VCI when analysing large, compared to small volumes. This makes direct comparisons of results between EBRT (typically large volumes) and BT (typically small volumes) studies challenging. In addition, the possibility to directly compare studies is limited due to differences in formalisms applied and the number of observers/cases analysed.^51^–^53^ Nevertheless; our results indicate that the implementation of 3D MRI for contouring introduced no volumetric uncertainties when compared to the conventional approach.

VCI gives no information on the absolute magnitude of contouring variations and their topography. However, brachytherapy is characterized by an inhomogeneous dose distribution with a steep dose gradient. Consequently, the magnitude and topography of contouring uncertainties have a direct impact on treatment. In our study, quantitative assessment of variability revealed mean distances of up to 2.8 (SD 0.9) mm and 2.7 (SD 0.7) mm between analysed pairs of contours for the middle and cranial parts of the HR CTV, respectively. In caudal parts of the contoured volumes, mean inter-approach and inter-observer distances between delineations of up to 3.7 (SD 2.1) and 5.4 (SD 3.1) mm, respectively, were found (Table 2, Figures 2, 3). Therefore, higher variability was present in those parts of the HR CTV, which are typically located in the close proximity of the ring source-channel. It has to be noted that up to approximately 4% deviation of commonly reported DVH parameters for the HR CTV can be expected per mm relative displacement of the contours with respect to the applicator.^58^ In addition, relatively large standard deviations of inter-contour distances reflect a considerable spread of individual values, in particular in the caudal regions of the HR CTV (Table 2, Figures 2, 3). Assessment of the effects of these variations on the commonly evaluated dose-volume parameters was not a subject of our investigation and will deserve special attention in the future. Contouring variation should be considered as one of the most important sources of uncertainties in IGABT.

Due to a 1 mm isotropic voxel size, 3D MRI allowed for a high-resolution image resampling in any plane within the TPS (Figure 1B). In this way, the need for an additional DICOM viewer during delineation could be omitted, reducing the infrastructural requirements of the department and facilitating contouring.

There are some limitations of our study. We evaluated the capability of 3D MRI to replace conventional 2D multi-planar MRI for delineation of HR CTV. The value of this approach for contouring of the gross tumour volume and intermediate risk CTV was not assessed. However, since the HR CTV is currently the most widely used volume for dose prescription and optimisation in IGABT, our results can be considered practically relevant.^20^,^22^,^23^,^29^ Next, since the inter-observer analysis was chosen to assess the inter-approach variations it could be argued that the number of observers (2) may be suboptimal and that the reported differences in inter-observer comparison could be attributed to the variability caused by particular case and not the observer. However, due to the specific expertise, required for the competence of a radiation oncologist in the field of cervix cancer IGABT, it is very challenging to obtain a higher number of observers for this purpose in a mono-institutional setting. Our results should be regarded as an indication of the value of the 3D MRI in gynaecological IGABT. However, further studies with higher number of observers may be required to confirm this. Finally, the SPACE sequence is vendor-specific. Before extrapolating the findings of our study to sequences from other vendors, additional work may be required.

It has to be noted that the pixel dimensions are larger on the para-transverse images, resampled from the 3D MRI (1 × 1 mm) when compared to the conventional para-transverse 2D MRI (0.6 × 0.6 mm). This results in a lower resolution and image quality in the main contouring plane. Eventual improvements of the 3D MRI are expected to further enhance the capability to use these sequences for treatment planning. Nevertheless, our results demonstrate that the described limitation did not result in an increase of contouring uncertainties.

Potential impact of MRI characteristics on the applicator reconstruction uncertainties deserves special mention. The procedure of defining the source channels on the images is a crucial step in IGABT, since the dose calculation is based on the geometry of source positions.^59^ Imaging with small slice thickness or the use of 3D sequences with isotropic voxel size has been recommended by the Gyn GEC-ESTRO working group to reduce the applicator reconstruction uncertainties. In addition, contouring and reconstruction should be performed preferably in the same image series to avoid fusion uncertainties.^30^ 3D MRI, evaluated in our study, therefore seems particularly suitable for gynaecological IGABT. Due to a small voxel size it enables an accurate applicator reconstruction, while the uncertainties related to image registration and fusion (Figure 1C) are avoided by using a single sequence for contouring and applicator reconstruction.^31^

## Conclusions

3D MRI-based contouring of HR CTV introduced no systematic deviations from the conventional 2D multi-planar MRI-based approach. By omitting multi-planar MRI and employing a single 3D sequence for treatment planning, total image acquisition time was shortened for more than 50 %, when compared to our conventional technique. In addition, uncertainties due to patient motion between sequences were reduced. The use of additional DICOM viewer was no longer required during delineation, facilitating the contouring process. By employing a single 3D MRI sequence with 1 mm isotropic voxel size both for contouring and applicator reconstruction, uncertainties of image registration and fusion are avoided and the applicator reconstruction facilitated. 3D MRI could be considered as an alternative to conventional 2D multi-planar MRI in cervix cancer IGABT, making this treatment method potentially more widely employed.

## Figures and Tables

**TABLE 1:** Inter-approach and inter-observer differences in HR CTV sizes

	**Diff. in HR CTV [cm**^3^**] (mean (SD))**	**Limits of agreement (mean +/− 2SD) [cm**^3^**]**	**95% CI [cm**^3^**]**	**CCC**	**95% boot CI**
**Inter-approach analysis**					
Observer 1	−1.2 (2.3)	−5.8, 3.4	−8.0, 5.7	0.97	0.94, 0.99
Observer 2	1.4 (2.8)	−4.3, 7.1	−7.1, 10.0	0.96	0.88, 0.99
**Inter-observer analysis**					
Conventional approach	2.8 (4.5)	−6.2, 11.9	−10.8, 16.4	0.87	0.75, 0.96
Test approach	0.3 (4.5)	−8.7, 9.2	−13.2, 13.7	0.91	0.84, 0.96

SD = standard deviation of differences; 95% CI = limits of 95% confidence interval for lower and upper limit; CCC = Lin’s concordance correlation coefficient; 95% boot CI = 95% bootstrap confidence interval for CCC

**TABLE 2:** Inter-approach and inter-observer distances between contours at different cranio-caudal levels of the HR CTV

	**Inter-contour distance [mm] (mean (SD))**	**Limits of agreement [mm]**	**95% CI [mm]**
	**All slices**		
**Inter-approach analysis**			
Observer 1	2.6 (0.4)	1.8, 3.4	1.3, 3.8
Observer 2	2.8 (0.7)	1.5, 4.2	0.8, 4.9
**Inter-observer analysis**			
Conventional approach	3.1 (0.8)	1.5, 4.7	0.8, 5.5
Test approach	3.0 (0.7)	1.5, 4.5	0.8, 5.2

	**Caudal two slices**		

**Inter-approach analysis**			
Observer 1	3.5 (1.5)	0.5, 6.5	−1.0, 8.0
Observer 2	3.7 (2.1)	−0.5, 8.0	−2.6, 10.1
**Inter-observer analysis**			
Conventional approach	5.4 (3.1)	−0.9, 11.7	−4.0, 14.9
Test approach	4.4 (3.0)	−1.7, 10.5	−4.7, 13.5

	**Middle slices**		

**Inter-approach analysis**			
Observer 1	2.4 (0.3)	1.7, 3.0	1.7, 3.3
Observer 2	2.8 (0.9)	0.9, 4.7	0.0, 5.6
**Inter-observer analysis**			
Conventional approach	2.6 (0.4)	1.7, 3.5	1.3, 3.9
Test approach	2.5 (0.7)	1.1, 4.0	0.4, 4.7

	**Cranial two slices**		

**Inter-approach analysis**			
Observer 1	2.1 (0.5)	1.1, 3.2	0.6, 3.7
Observer 2	2.4 (0.8)	0.7, 4.1	−0.1, 4.9
**Inter-observer analysis**			
Conventional approach	2.5 (1.0)	0.5, 4.5	−0.5, 5.5
Test approach	2.7 (0.7)	1.3, 4.0	0.6, 4.7

SD = standard deviation of differences; 95% CI = limits of 95% confidence interval for lower and upper limit.
